# Work-related socioeconomic determinants of health: evidence from educational mismatch in Italy

**DOI:** 10.3389/fpubh.2024.1388093

**Published:** 2024-07-24

**Authors:** Cristiana Abbafati, Aldo Rosano

**Affiliations:** ^1^Department of Juridical and Economic Studies, Sapienza University of Rome, Rome, Italy; ^2^Department of Civil Economy and Migration Processes, Istituto nazionale per l'analisi delle politiche pubbliche (INAPP), Rome, Italy

**Keywords:** educational mismatch, health equity, gender gap, work related socioeconomic determinant, occupation

## Abstract

**Introduction:**

An educational mismatch is defined as the situation where the education qualifications of an employee do not match the qualifications required for the job they do. A mismatch can be vertical where the level of the employee's qualification is not the one required by the job. This study contributes to the literature on work-related social determinants of health, by carrying out the first assessment of the relationship between educational mismatch and health in Italy.

**Methods:**

Data come from PLUS, a national survey of labor supply. The risk of suffering from bad or very bad health associated with educational mismatch is investigated through a logistic regression model accounting for the socioeconomic context and occupation.

**Results:**

Our findings show women are at greater risk of suffering from bad or very bad health than men, especially if under-educated.

**Discussion:**

Our results show the need to address more research on work-related social determinants of health, which can represent a barrier to achieving health equity.

## 1 Introduction

An educational mismatch is defined as the situation where the education qualifications of an employee do not match the qualifications required for the job they do. A mismatch can be vertical where the level of the employee's qualification is not the one required by the job. Since Duncan and Hoffman's seminal work (1), educational mismatch has been extensively debated for the negative consequences that it may produce on labor markets. Originating from characteristics of both the supply and demand of labor (2), it affects wages (3–7), productivity (8), career mobility (9–11), and job satisfaction (12, 13). Educational mismatch is also viewed as an important public health concern. Several studies associate it with negative health outcomes, especially mental health conditions (14–21). This association pertains to what is called “status inconsistency” (22), i.e., “a discrepancy between the position a person holds in one domain of their social environment comparative to their position in another domain” (15). The remaining, scarce literature focuses on mortality, injuries, and self-assessed health (23–27).

Most existing empirical studies on educational mismatch concentrate on advanced economy countries, where this phenomenon is most evident, and investigate its effects both on the labor market and health outcomes. To our knowledge, studies regarding Italy are focused only on the labor market (10, 28), which suffers from long-term disequilibrium across regions, marked by the country's historic and persistent North-South developmental gap (29–32). The interactions between macroeconomic events and institutional configurations (33) result in high structural unemployment with age, gender, and sectoral disparity, further worsened by the Great Recession. In addition, Italy exhibits three other peculiar traits: the highest percentage of young people not in employment, education, or training (NEETs), the lowest attendance in tertiary education and the second lowest women's employment rate in Europe (34). However, the Italian situation bears particular interest with regard to the possible relationship between educational mismatch and health: high percentages of educational mismatch in the Italian labor market (35) coexist with long, and healthy life expectancy, which is one of the highest in the world, a low fertility rate and aging workers with increasing number of years lived with disability (36). Studies of educational mismatch and health have focused mainly on the over-educated for which investments in education do not match their occupational position (37, 38), clearly showing the “status inconsistency.” Educational mismatch comprise over-qualification, under-employment, and occupational downgrading (27) and can cause experience of relative deprivation and ultimately bad health (14, 16, 26, 27, 39). Similarly, the under-educated may be more likely to experience relative deprivation and associated stress or poor health effects when they compare themselves to others with similar levels of attained education but highest income or occupational status (27). This study aims at contributing to the literature on work-related social determinants of health, by carrying out the first ever assessment of the relationship between vertical educational mismatch and health for the over- and under-educated workers in Italy.

## 2 Data and methods

### 2.1 Data source

Data come from the Participation Labor Unemployment Survey (PLUS) (40) by the National Institute for Public Policy Analysis (INAPP). PLUS is an Italian national survey periodically conducted since 2005. It is included in the National Statistical Plan and is part of the official statistics network (SISTAN). The 2021 edition is based on a sample of 46,262 participants aged 18–74 years old and has been conducted between March and July 2021. The participation rate in the 2021 edition was 56.3%. PLUS adopted a stratified quota sampling with partially overlapping planned domains. Stratification was implemented considering the following conditions: region of residence, type of municipality, sex, age, and employment status. The questionnaire counts a total of about 200 questions. Individuals were interviewed by telephone through a Computer-Assisted Telephone Interviewing (CATI) technique and in the absence of proxy respondents, i.e., the answers were given directly and exclusively by the interviewee, thereby reducing measurement errors and partial non-responses. PLUS has a longitudinal structure, providing details about some social and economic indicators such as work and family income, education, and health and it is aimed mainly at investigating problematic aspects of the labor market through modules for each contractual typology and sub-modules dedicated to specific topics, allowing to read phenomena at different levels.

### 2.2 Selection of variables and description

One of the specific modules available in PLUS concerns educational mismatch measured by worker's self-assessment, one of three methods generally used for quantifying educational mismatch together with job analysis and realized matched (41). The subjective definition of educational mismatch results from the “What level of education do you think is most appropriate for your job position?” question. Based on the worker's response, a vertical educational mismatch is noted by comparing the worker's actual education to that which they believe (self-reported) to be the appropriate education requirements for their job position (5). The term “over-educated” is used to describe individuals who have attained a level of education that is higher than that which they believe to be appropriate for their job role. Conversely, the term “under-educated” is used to describe individuals who have attained a level of education that is lower than that which they believe to be appropriate for their job role.

Additionally, PLUS dedicates a specific module on perceived health. The question is “How do you judge your overall health?” and the respondent has five possible answers corresponding to: very good, good, fair, bad, and very bad perceived health.

Occupation, coded according to ISCO-88 classification, is also investigated in PLUS and hence considered in the analysis. Finally, PLUS allows to detect the socioeconomic context, through the following variables: sex, age, marital status, having children, geographical location, and urbanization. As well-known, sex is one of the discriminating factors influencing the opportunities to obtain a job (42, 43). In Italy, despite the fact that women are often more educated than men they experience a higher percentage of unemployment, more frequent part-time jobs, and lower wage growth than males (44, 45). Women's participation depends on their partners' labor position, institutional context, welfare measures and relative position in the labor market (46, 47). Although, women live longer than men, their health perception is generally worse than men (48). Age is another key factor. Italy is experiencing two simultaneous phenomena: an aging workforce and a low rate of youth employment, one of the lowest in Europe (34). Typically, youth have better health than adults; however, given that youth employment is more sensitive to business cycles (49), a sense of frustration may arise, negatively affecting the perception of health. The more children's women have, the greater the gap in female and male employment rates in Italy (44). Furthermore, although some studies show that having children protects female health (50, 51), care for children may result in increasing stress both for men and women, and often lead them to neglect their health problems. Being married is recognized as a protective factor for health. It positively affects mental health (52), mortality (53, 54), and healthy behaviors (55, 56). Geographical location highlights Italy's historical North-South dualism regarding education, employment, and health. Urbanization is considered as well, since one third of the Italian territory is mountainous. Mountain municipalities, with a few thousand inhabitants, are almost half of the total of Italian municipalities (44). They suffer from marginality (57), shortage of local social and health services, and reduced hospital facilities (58).

### 2.3 Analytical strategy

The PLUS survey is representative of the Italian working-age population. Nineteen thousand and twenty-five subjects who declared to be occupied were included in the study. Data were analyzed applying weights to adjust the results for the sample design and to provide frequency distributions representing the target population (e.g., employed population). Such weights were calculated adopting methodologies based on generalized regression estimators (59). Descriptive statistics were used to analyse the distribution of age, sex, and area of residence of workers by education; age, sex, area of residence and education by perceived health status; age, sex, area of residence, education, urbanization level, marital status, and childbearing by type of mismatch. The crude and age standardized percentages of workers suffering from bad health by occupation, educational mismatch, and sex were also calculated. Data were analyzed according to a cross-sectional design.

The risk of suffering from bad or very bad health (the outcome variable) associated with educational mismatch (exposure variable) was investigated through a logistic regression model accounting for the socioeconomic context, considering sex (males/females), age (18–29/30–49/50–74 years), marital status (single/married/separated or divorced/widowed), having children (yes/no), geographical location (North/Center/South), urbanization (up to 5,000 residents/from 5,000 to 10,000 residents/from 10,000 to 30,000 residents/from 30,000 to 100,000 residents/from 100,000 to 250,000 residents/more than 250,000 residents), and nine macro-categories of occupation (administrative workers/intellectual scientific and highly skilled professionals/professionals/clericals and related workers/service workers/craft workers/production and related workers/unskilled workers/armed forces) as potential confounding factors. In the logistic models standardized weights, to sum to the sample size, were used to avoid spurious significance due to an “inflated” sample size. In order to analyse the role of EM in the relationship between sex and perceived health an interaction term was included in the regression models. Analyses were performed with “Stata 17” (60). All odds ratios (ORs) are given with 95% confidence intervals (CIs).

## 3 Results

### 3.1 Descriptive statistics

Almost 50% of all the respondents have a secondary level of education (high school) while about 30% have a degree (University degree). Women are better educated than men. Respondents with higher education are more frequent in the South of Italy and in the age class 30–49 years (Supplementary Table 1).

Regarding health, self-assessed health is high-grade overall. Only 6.3% of men and 9.4% of women perceived bad or very bad health. If only slightly, women feel worse than men, as we expected. There are no major differences between education levels, except for those who have primary schooling and report more feelings of bad or very bad health. There are no substantial dissimilarities between areas of the country, except for those residents of the South who report feeling bad health (Supplementary Table 2).

More than half of the sample respondents, corresponding to 69.6%, considered their level of education appropriate for their jobs, while 18.5% think they were over-educated and 11.9% under-educated (Supplementary Figure 1).

Men suffer from educational mismatch more than women and under-educated men are more than over-educated. Observing age, the majority of the over-educated, are aged 30–49 while the age range 50–75 prevails among the under-educated. Both over- and under-educated, 49.1 and 57.6% in that order, are prevalent in the North and live in a medium dimension of urbanization. Finally, the over-educated are single and without children, while the under-educated are married with children (Supplementary Table 3).

Relative to categories of occupation, the highest percentage of over-educated men is noted in “Industrial plant operators” and “Unskilled professions in manufacturing, mineral extraction and construction while over-educated women in “Farmers and skilled laborers in agriculture, forestry, animal husbandry, fishing, and hunting.” For under-educated men the highest percentage is in “Unskilled professions in agriculture, grounds maintenance, animal husbandry, forestry, and fishing” while under-educated women in “Semi-skilled workers of fixed machinery for mass production and assembly workers” (for data on other professions, see Supplementary Table 4). It is crucial to acknowledge that the holding a specific profession and experiencing an educational mismatch is influenced by the fact that the latter is self-reported and, most importantly, reflects expectations about one's life that may not be entirely realistic. We delve deeper into this in the Limitation section.

**Figure 1:**
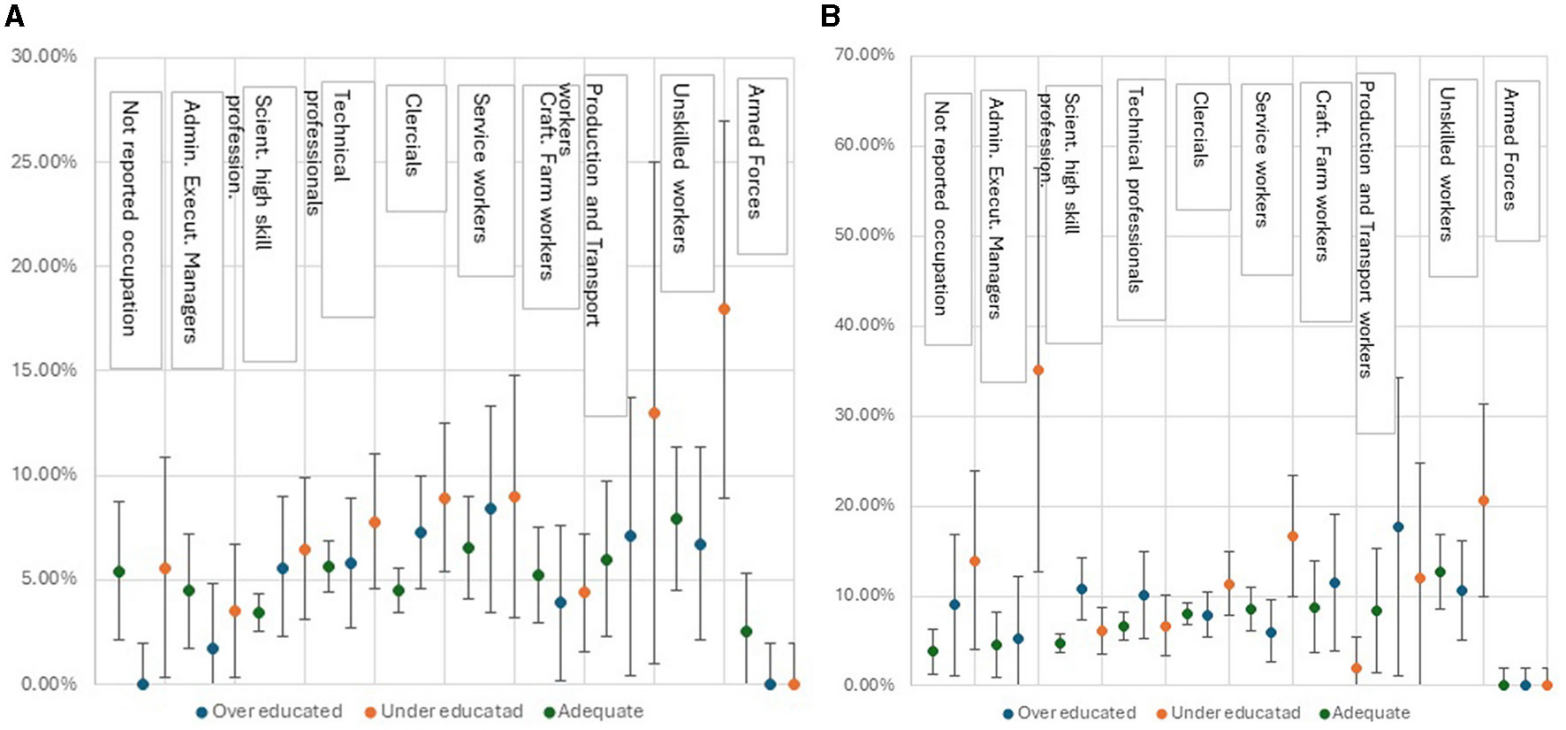
**(A)** Prevalence of bad and very bad health perception by type of educational mismatch for macro professional categories: adjusted percentage by age with 95% Confidence Intervals (C.I.)—males. **(B)** Prevalence of bad and very bad health perception by type of educational mismatch for macro professional categories: adjusted percentage by age percentages with 95% Confidence Intervals (C.I.)—females.

The under- and over-educated in Italy perceive a good health status overall. Only 7.6% of respondents say they suffer from bad or very bad health and the over-educated suffer a little less than the under-educated (Supplementary Figure 2). Among the 9-macro occupational macro-categories the perception of poor health (bad and very bad health) for over-educated men, is more frequent, compared to workers with adequate education, in “Clerical and Related Workers” and “Service Workers and Shop and Market Sales Workers” categories, even though not in a statistically significant way. While for under-educated is in “Production and Related Workers, Transport Equipment Operators and Laborers” and “Unskilled workers.” Regarding women, a gender disadvantage is confirmed: under-educated women suffer more than men in almost all occupational categories observed, significantly among “Administrative. Executive and Managerial Workers” and “Service workers” (Figures 1A, B).

A detailed analysis of the 38 occupations across the 9 categories reveals that overeducated men who report perceived poor health are employed in in “Secretarial and office machine clerks,” “Skilled professions in commercial activities” and “Unskilled occupations in domestic, recreational and cultural activities” while the under-educated in “Technical professions in public and personal services,” “Secretarial and office machine clerks” and “Unskilled occupations in trade and services.” On the other hand, the occupations in which women report more likely poor health if they are over-educated are “Engineers, architects and assimilated professions,” “Specialists in the life sciences” and “Education and research specialists” while those under-educated in “Secretarial and office machine clerks,” “Skilled professions in cultural, security and personal services” and “Unskilled occupations in domestic, recreational and cultural activities” (for details regarding perceived and self-reported ill health in all 38 occupations see Supplementary Tables 5, 6).

### 3.2 Findings from the logistic regression

The analysis conducted through the logistic regression model showed different patterns for the under- and over-educated workers, with significantly higher risks for under-educated (OR = 1.41, 95% C.I. = 1.17–1.70) compared to workers with adequate education, and no significant difference for over-educated (OR = 0.89, 95% C.I. = 0.72–1.11). The probability of suffering from bad or very bad health was significantly higher for women (OR = 1.57, 95% C.I. = 1.38–1.79), adults and older, separated/divorced or widowed, and those residing in the South. The interaction between sex and EM revealed an increased risk among female workers, both under-educated and overeducated, even though not statistically significant. Among other adjusting factors, the level of urbanization showed an increased risk only for cities from 100,000 and 250,000 residents, as well as professions like “Service workers” (OR = 1.95, 95% C.I. = 1.33–2.87), “Production workers” (2.49, 95% C.I. = 1.56–3.97), and “Unskilled workers” (2.63; 95% C.I. = 1.77–3.91; Table 1).

**Table 1:** Risk of suffering, in terms of odds ratios (OR), from bad and very bad health associated with the educational mismatch (EM) for over-and under-education, adjusted for potential confounders with 95% confidence intervals (C.I.).

**Socio-demographic characteristics**			
**Mismatch**	ORs	95% CIs	
Adequate education (ref)	1		
Over educated	0.89	0.72	1.11
Under educated	1.41	1.17	1.70
**Sex**			
Male (ref)	1		
Female	1.57	1.38	1.79
**Age**			
18–29 (ref)	1		
30–49	2.83	2.18	3.66
50–74	3.71	2.85	4.85
**Area**			
North (ref)	1		
Center	0.78	0.68	0.89
South	1.33	1.17	1.51
**Urbanization**			
Up to 5,000 residents (ref)	1		
From 5,000 to 10,000 res.	0.90	0.73	1.10
From 10,000 to 30,000 res.	0.95	0.80	1.13
From 30,000 to 100,000 res.	1.06	0.89	1.25
From 100,000 to 250,000 res.	1.24	1.03	1.50
More than 250,000 res.	1.08	0.90	1.30
**Marital status**			
Single (ref)	1		
Married	0.99	0.85	1.14
Separated/divorced	1.42	1.14	1.75
Widowed	1.52	1.01	2.30
**Having children**			
Yes (ref)	1		
No	0.98	0.86	1.12
**Occupation**			
Administrative. Executive and managerial workers (ref)	1		
Intellectual. Scientific and highly skilled professionals	1.17	0.81	1.69
Professional. Technical and related workers	1.60	1.11	2.31
Clerical and related workers	1.60	1.12	2.30
Service workers and shop and market sales workers	1.95	1.33	2.87
Craft. Farm and skilled workers	1.58	1.03	2.42
Production and related workers. Transport equipment operators and laborers	2.49	1.56	3.97
Unskilled workers	2.63	1.77	3.91
Armed forces	0.36	0.12	1.13
**Interaction educational mismatch (EM)** ^*^ **sex**			
EM (overeducated) ^*^ females	1.24	0.93	1.65
EM (undereducated) ^*^ females	1.10	0.84	1.44

## 4 Discussion

There are about 7 million Italian workers who perceive a mismatch between the educational qualification they attained and the one appropriate for their job. Of these, nearly 3 million are women, the majority of them are over-educated. The probability of suffering from bad health is significantly higher among under-educated workers confirming the well-known relationship between education and health (61).

Concerning the role of gender in the relationship between educational mismatch and health, this study showed an interaction effect, even though not statistical significant, between EM and gender, with a clear disadvantage for female workers, and a higher percentage of over-educated female workers in some professions, such as “Specialists in mathematical, computer, chemical, physical and natural sciences” “Engineers, architects, and assimilated professions” and “Specialists in life sciences,” suffering from poor health. These findings suggest the possibility of a higher susceptibility of female workers with EM to perceiving bad health. Even though the estimated risks did not reach the statistical significance, the pattern is quite evident: the risk of suffering from bad health, in terms of OR, of under-educated women is 2.43 (calculated as the combination of the two conditions—being a female and under educated worker—and the estimated interaction term, estimated using as the reference category that of men with no educational mismatch) and, similarly, the risk for over-educated is 1.73. Our results are entirely consistent with findings in the literature, particularly those that have highlighted inefficiencies and inequalities in labor market functioning in certain countries (26). They also are consistent with Italian labor market data on the gender disadvantage of women relative to men in the labor market (44), especially if they are separated/divorced or widowed.

Our results are also consistent with evidence in this regard concerning age (36). The risk of perceived bad or very bad health status increases if a woman is an adult or older adult. What is more, we confirm what is already known with respect to the Italian historic and persistent North-South developmental gap for health, education, and work (62–64) and for which Italian women residing in the South have a higher risk of perceiving bad or very bad health. The mechanisms through which educational mismatch can have repercussions on health can be multiple and trace back to the effects such a mismatch has on mental health, but this may also depend on the family, social and general context (20). Indeed, in the South, more than elsewhere, achieving effective universal health coverage with equity is compounded by social and economic deprivation, inefficient public services, environmental damage, unemployment and even crime and it is well-known how heavily socioeconomic context affects health (65).

Another related factor affecting workers' health is occupation. “Unskilled workers” have a higher risk of suffering from poor health than the other categories, as well as “Service Workers and Shop and Market Sales Workers” and “Production workers.” A gender health disadvantage is also confirmed in this: under-educated women suffer more than men in almost all occupational categories observed.

In contrast, no association was found for the other socioeconomic variables included in the analysis. In Italy, having children is not associated with bad or very bad health neither living in a city, with the sole exception of living in larger cities.

### 4.1 Strengths and limitations

The first strength is that this study is on an issue never previously investigated for Italy. It makes an important contribution by providing new insights into work-related determinants of health, especially for women at greater risk of suffering from bad or very bad health than men. The second strength deals with the sample size that allows to analyze the occupational groups in details: the absolute number of several sampled workers by occupational groups is never <30 that constitutes a valid sample size.

Concerning the limitations, the first relies on self-assessed measures. They have often been criticized for their biases and misinterpretation due to various factors, including individual personality traits, which can lead to perceived mismatch and poor health (7, 46, 66, 67). The second limitation rests on the cross-sectional analysis, which, by nature, does not allow one to verify a causal relationship between educational mismatch and health. Finally, a possible selection bias could be happened because of a limited participation rate (56.3%), which is, on the other hand, in line with the participation rate of other similar national large survey (68).

## 5 Conclusions

This study has examined the relationship between educational mismatch and health in Italy. Both descriptive and empirical analysis, based on cross-sectional data from PLUS 2021 (40), seem to indicate a possible association between vertical educational mismatch and bad or vary bad health, especially for Italian under-educated women. Our study provides reliable hints on the health burden estimation of workers with EM suffering from bad health. Even though the study did not provide conclusive results, the findings constitute an important contribution by providing insights into an issue never investigated for Italy and emphasize the need to address more research on work-related social determinants of health which can represent a barrier to achieving health equity. Future developments may involve using the panel aspect of PLUS and investigating the relationship between skill mismatch and health considering several potential confounding factors.

## Data availability statement

Original datasets are available in a publicly accessible repository: INAPP, PLUS dataset: https://oa.inapp.org/xmlui/handle/20.500.12916/951.

## Author contributions

CA: Conceptualization, Formal analysis, Funding acquisition, Investigation, Methodology, Project administration, Resources, Supervision, Validation, Visualization, Writing – original draft, Writing – review & editing. AR: Conceptualization, Data curation, Formal analysis, Investigation, Methodology, Software, Supervision, Validation, Visualization, Writing – original draft, Writing – review & editing.
