# Supplementary material for: Work-related socioeconomic determinants of health: evidence from educational mismatch in Italy
Source: Front Public Health. 2024 Jul 24;12:1388093. doi: 10.3389/fpubh.2024.1388093 (PMC11303326; doi:10.3389/fpubh.2024.1388093)

**Supplementary Materials**

**Table A1. Education by sex, age class, and area of residence**

|  | Primary (Elementary and Low Secondary School) | | Secondary (High School) | | Degree (University Degree) | | Post-degree (Post-University Degree) | |
| --- | --- | --- | --- | --- | --- | --- | --- | --- |
|  | n | % | n | % | n | % | N | % |
| **Sex** |  |  |  |  |  |  |  |  |
| Male | 805,049 | 6.1% | 6,565,099 | 49.7% | 4,619,651 | 35.0% | 1,220,495 | 9.2% |
| Female | 531,995 | 5.5% | 4,398,905 | 45.8% | 3,626,485 | 37.8% | 1,045,497 | 10.9% |
| **Age** |  |  |  |  |  |  |  |  |
| 18-29 | 113,547 | 4.2% | 1,625,206 | 60.7% | 838,126 | 31.3% | 102,502 | 3.8% |
| 30-49 | 443,909 | 3.9% | 4,719,456 | 41.5% | 4,760,351 | 41.8% | 1,457,717 | 12.8% |
| 50-75 | 779,588 | 8.9% | 4,619,342 | 52.8% | 2,647,659 | 30.3% | 705,773 | 8.1% |
| **Area** |  |  |  |  |  |  |  |  |
| North | 788,043 | 6.6% | 5,891,454 | 49.5% | 4,149,404 | 34.9% | 1,061,670 | 8.9% |
| Center | 263,866 | 5.4% | 2,251,174 | 46.1% | 1,801,057 | 36.9% | 564,800 | 11.6% |
| South | 285,135 | 4.7% | 2,821,376 | 46.7% | 2,295,675 | 38.0% | 639,522 | 10.6% |

**Table A2. Perceived health status by sex, age, education, and area of residence (crude percentages)**

|  | Very good | | Good | | Fair | | Bad and Very Bad | |
| --- | --- | --- | --- | --- | --- | --- | --- | --- |
|  | N | % | N | % | n | % | n | % |
| **Sex** |  |  |  |  |  |  |  |  |
| Male | 2,253,893 | 17.10% | 6,796,663 | 51.40% | 3,325,563 | 25.20% | 834,175 | 6.30% |
| Female | 1,401,745 | 14.60% | 4,956,721 | 51.60% | 2,343,783 | 24.40% | 900,633 | 9.40% |
| **Age** |  |  |  |  |  |  |  |  |
| 18-29 | 1,089,196 | 40.70% | 1,195,082 | 44.60% | 322,845 | 12.00% | 72,258 | 2.70% |
| 30-49 | 1,782,456 | 15.70% | 6,077,571 | 53.40% | 2,733,979 | 24.00% | 787,427 | 6.90% |
| 50-75 | 783,986 | 9.00% | 4,480,731 | 51.20% | 2,612,522 | 29.80% | 875,123 | 10.00% |
| **Education** |  |  |  |  |  |  |  |  |
| Primary (Elementary and Low Secondary School) | 177,668 | 13.30% | 611,137 | 45.70% | 408,797 | 30.60% | 139,442 | 10.50% |
| Secondary (High School) | 1,671,958 | 15.20% | 5,549,028 | 50.60% | 2,786,293 | 25.40% | 956,725 | 8.80% |
| Degree (University Degree) | 1,437,755 | 17.40% | 4,414,496 | 53.50% | 1,881,972 | 22.80% | 511,913 | 6.20% |
| Post-degree  (Post-University Degree) | 368,257 | 16.30% | 1,178,723 | 52.00% | 592,284 | 26.10% | 126,728 | 5.60% |
| **Area** |  |  |  |  |  |  |  |  |
| North | 2,013,221 | 16.90% | 6,304,689 | 53.00% | 2,687,388 | 22.60% | 885,273 | 7.50% |
| Center | 797,272 | 16.30% | 2,550,662 | 52.30% | 1,239,352 | 25.40% | 293,611 | 6.00% |
| South | 845,145 | 14.00% | 2,898,033 | 48.00% | 1,742,606 | 28.80% | 555,924 | 9.20% |

**Table A3. Socio-demographic characteristics of interviewed subjects by type of educational mismatch (column percentages)**

|  | Appropriate | | Over-educated | | Under-educated | | Total | |
| --- | --- | --- | --- | --- | --- | --- | --- | --- |
|  | n | % | n | % | n | % | n | % |
| **Sex** |  |  |  |  |  |  |  |  |
| Male | 9,025,708 | 68.32% | 2,161,648 | 16.36% | 2,022,938 | 15.31% | 13,210,294 | 100.00% |
| Female | 6,651,451 | 69.27% | 1,786,284 | 18.60% | 1,165,147 | 12.13% | 9,602,882 | 100.00% |
| **Education** |  |  |  |  |  |  |  |  |
| Primary (Elementary and Low Secondary School) | 779,726 | 58.32% | 0 | 0.00% | 557,318 | 41.68% | 1,337,044 | 100.00% |
| Secondary (High School) | 7,481,642 | 68.24% | 1,269,380 | 11.58% | 2,212,982 | 20.18% | 10,964,004 | 100.00% |
| Degree (University Degree) | 6,315,636 | 76.59% | 1,512,715 | 18.34% | 417,785 | 5.07% | 8,246,136 | 100.00% |
| Post-degree (Post-University Degree) | 1,100,155 | 48.55% | 1,165,837 | 51.45% | 0 | 0.00% | 2,265,992 | 100.00% |
| **Age** |  |  |  |  |  |  |  |  |
| 18-29 | 1,944,208 | 72.56% | 534,226 | 19.94% | 200,947 | 7.50% | 2,679,381 | 100.00% |
| 30-49 | 7,784,106 | 68.39% | 2,302,556 | 20.23% | 1,294,771 | 11.38% | 11,381,433 | 100.00% |
| 50-75 | 5,948,845 | 67.97% | 1,111,150 | 12.70% | 1,692,367 | 19.34% | 8,752,362 | 100.00% |
| **Perceived health status** |  |  |  |  |  |  |  |  |
| Very good | 2,611,400 | 71.43% | 703,698 | 19.25% | 340,540 | 9.32% | 3,655,638 | 100.00% |
| Good | 8,178,278 | 69.58% | 1,944,694 | 16.55% | 1,630,412 | 13.87% | 11,753,384 | 100.00% |
| Fair | 3,797,545 | 66.98% | 1,011,054 | 17.83% | 860,747 | 15.18% | 5,669,346 | 100.00% |
| Bad | 963,646 | 65.11% | 221,502 | 14.97% | 294,816 | 19.92% | 1,479,964 | 100.00% |
| Very bad | 126,290 | 49.56% | 66,984 | 26.28% | 61,570 | 24.16% | 254,844 | 100.00% |
| **Area** |  |  |  |  |  |  |  |  |
| North | 8,115,212 | 68.25% | 1,939,425 | 16.31% | 1,835,934 | 15.44% | 11,890,571 | 100.00% |
| Center | 3,343,166 | 68.49% | 906,568 | 18.57% | 631,163 | 12.93% | 4,880,897 | 100.00% |
| South | 4,218,781 | 69.83% | 1,101,939 | 18.24% | 720,988 | 11.93% | 6,041,708 | 100.00% |
| **Urbanization** |  |  |  |  |  |  |  |  |
| up to 5,000 residents | 2,533,198 | 68.05% | 619,756 | 16.65% | 569,525 | 15.30% | 3,722,479 | 100.00% |
| from 5,000 to 10,000 | 1,830,804 | 67.37% | 452,535 | 16.65% | 434,252 | 15.98% | 2,717,591 | 100.00% |
| from 10,000 to 30,000 res. | 3,212,355 | 68.65% | 817,641 | 17.47% | 649,052 | 13.87% | 4,679,048 | 100.00% |
| from 30,000 to 100,000 res. | 3,733,255 | 70.71% | 858,969 | 16.27% | 687,634 | 13.02% | 5,279,858 | 100.00% |
| from 100,000 to 250,000 res. | 1,841,446 | 67.68% | 518,650 | 19.06% | 360,841 | 13.26% | 2,720,937 | 100.00% |
| over 250,000 res. | 2,526,101 | 68.40% | 680,381 | 18.42% | 486,781 | 13.18% | 3,693,263 | 100.00% |
| **Marital status** |  |  |  |  |  |  |  |  |
| Single | 6,000,405 | 68.43% | 1,861,469 | 21.23% | 907,057 | 10.34% | 8,768,931 | 100.00% |
| Married | 8,673,066 | 69.54% | 1,833,281 | 14.70% | 1,965,949 | 15.76% | 12,472,296 | 100.00% |
| Separated/Divorced | 865,386 | 63.76% | 218,939 | 16.13% | 272,934 | 20.11% | 1,357,259 | 100.00% |
| Widowed | 138,302 | 64.42% | 34,243 | 15.95% | 42,145 | 19.63% | 214,690 | 100.00% |
| **Childbearing** |  |  |  |  |  |  |  |  |
| Yes | 8,325,202 | 67.88% | 1,811,831 | 14.77% | 2,127,343 | 17.35% | 12,264,376 | 100.00% |
| No | 7,351,957 | 69.69% | 2,136,101 | 20.25% | 1,060,742 | 10.06% | 10,548,800 | 100.00% |

| **Table A4. Percentage of workers by Educational Mismatch, Gender and occupation** | |  |  |  |  |  |  |
| --- | --- | --- | --- | --- | --- | --- | --- |
|  | |  |  |  |  |  |  |
|  |  | Males and Females | | Males | | Females | |
| Cod | Profession | Over educated | Under educated | Over educated | Under educated | Over educated | Under educated |
| 1,1 | Members of legislative and governmental bodies, executives in public administration | 14.1% | 14.1% | 10.0% | 15.0% | 21.9% | 12.5% |
| 1,2 | Entrepreneurs, directors and managers of large companies | 14.0% | 18.9% | 12.3% | 18.0% | 19.0% | 21.4% |
| 1,3 | Entrepreneurs and managers of small businesses | 17.9% | 15.2% | 13.6% | 16.9% | 25.8% | 12.1% |
| 2,1 | Specialists in mathematical, computer, chemical, physical and natural sciences | 10.6% | 18.3% | 8.6% | 19.4% | 15.1% | 15.7% |
| 2,2 | Engineers, architects and assimilated professions | 13.1% | 6.8% | 10.8% | 7.3% | 17.4% | 5.8% |
| 2,3 | Specialists in the life sciences | 11.6% | 8.4% | 5.9% | 19.6% | 14.4% | 2.9% |
| 2,4 | Specialists in health care | 20.1% | 5.4% | 22.0% | 4.2% | 18.2% | 6.6% |
| 2,5 | Specialists in the humanities, social sciences, arts and management | 18.1% | 9.8% | 17.0% | 12.6% | 19.1% | 7.2% |
| 2,6 | Education and research specialists | 14.2% | 11.3% | 12.4% | 10.1% | 15.0% | 11.9% |
| 3,1 | Technical professions in science, engineering and manufacturing | 10.3% | 17.4% | 9.1% | 19.7% | 13.7% | 11.3% |
| 3,2 | Technical professions in organization, administration, and financial and business activities | 11.1% | 11.1% | 8.3% | 15.0% | 12.2% | 9.5% |
| 3,3 | Technical professions in health and life sciences | 13.0% | 14.4% | 12.4% | 15.6% | 13.7% | 12.9% |
| 3,4 | Technical professions in public and personal services | 16.4% | 14.2% | 15.2% | 14.3% | 17.3% | 14.0% |
| 4,1 | Secretarial and office machine clerks | 16.8% | 11.0% | 14.5% | 13.4% | 18.4% | 9.3% |
| 4,2 | Clerical clerks in money movement and customer service | 28.2% | 9.6% | 24.7% | 11.6% | 31.1% | 7.9% |
| 4,3 | Administrative, accounting and financial management clerks | 19.6% | 9.6% | 19.3% | 11.8% | 20.0% | 6.8% |
| 4,4 | Clerks employed in the collection, control, storage and delivery of documentation | 19.6% | 15.0% | 19.2% | 12.5% | 20.0% | 17.3% |
| 5,1 | Skilled professions in commercial activities | 30.3% | 9.6% | 21.1% | 12.5% | 36.4% | 7.6% |
| 5,2 | Skilled professions in accommodation and food service activities | 36.0% | 6.2% | 31.4% | 6.3% | 39.5% | 6.1% |
| 5,3 | Skilled professions in health and social services | 12.7% | 15.9% | 13.6% | 9.1% | 12.2% | 19.5% |
| 5,4 | Skilled professions in cultural, security and personal services | 21.8% | 11.6% | 17.6% | 12.3% | 24.6% | 11.1% |
| 6,1 | Skilled craftsmen and workers in food processing, wood, textiles, clothing, leather, and entertainment industries | 18.9% | 10.8% | 15.0% | 11.0% | 34.7% | 10.2% |
| 6,2 | Craft and skilled metalworkers and installers and maintenance workers of electrical and electronic equipment | 14.6% | 10.6% | 15.0% | 10.8% | 12.9% | 9.7% |
| 6,3 | Craft and skilled metalworkers in precision mechanics, artistic handicrafts, printing and related trades | 14.8% | 9.0% | 13.1% | 9.1% | 22.9% | 8.6% |
| 6,4 | Farmers and skilled laborers in agriculture, forestry, animal husbandry, fishing and hunting | 32.8% | 12.4% | 29.2% | 13.8% | 41.1% | 8.9% |
| 6,5 | Craftsmen and skilled workers in 'mining, construction and maintenance of buildings | 27.4% | 11.0% | 25.7% | 10.7% | 30.4% | 11.4% |
| 7,1 | Industrial plant operators | 40.0% | 6.7% | 41.7% | 6.7% | 36.7% | 6.7% |
| 7,2 | Semi-skilled workers of fixed machinery for mass production and assembly workers | 33.9% | 11.3% | 34.6% | 5.1% | 32.4% | 24.3% |
| 7,3 | Stationary machinery operators in agriculture and the food industry | 13.9% | 13.9% | 8.7% | 17.4% | 23.1% | 7.7% |
| 7,4 | Drivers of vehicles, mobile and lifting machinery | 19.3% | 14.3% | 18.2% | 14.7% | 27.8% | 11.1% |
| 8,1 | Unskilled occupations in trade and services | 32.1% | 13.8% | 29.7% | 15.2% | 34.6% | 12.2% |
| 8,2 | Unskilled occupations in domestic, recreational and cultural activities | 26.8% | 17.1% | 40.0% | 14.3% | 21.6% | 18.2% |
| 8,3 | Unskilled professions in manufacturing, mineral extraction and construction | 36.6% | 9.8% | 41.7% | 8.3% | 29.4% | 11.8% |
| 8,4 | Unskilled professions in agriculture, grounds maintenance, animal husbandry, forestry and fishing | 10.9% | 21.3% | 9.6% | 24.4% | 14.5% | 12.7% |
| 9,1 | Officers in the armed forces | 7.1% | 14.3% | 11.1% | 22.2% | 0.0% | 0.0% |
| 9,2 | Sergeants, superintendents and marshals of the armed forces | 22.9% | 11.4% | 25.0% | 12.5% | 0.0% | 0.0% |
| 9,3 | Troops of the armed forces | 18.9% | 8.9% | 16.2% | 8.8% | 27.3% | 9.1% |
|  | Total | 18.6% | 11.7% | 16.2% | 13.5% | 20.9% | 9.9% |

**Table A5. Crude and adjusted percentages, with 95% confidence interval (C.I.), of male workers in bad health by educational mismatch, gender and occupation**

|  |  | Percentage of Males in bad health /adequate education | | | | | Percentage of Males in bad health / over educated | | | | | Percentage of Males in bad health /under educated | | | | |
| --- | --- | --- | --- | --- | --- | --- | --- | --- | --- | --- | --- | --- | --- | --- | --- | --- |
| **Occupation** | Code | n (sample) | Crude percentages | Adjusted percentages | 95%C.I. lower limit | 95% C. I. upper limit | n (sample) | Crude percentages | Adjusted percentages | 95%C.I. lower limit | 95% C. I. upper limit | n (sample) | Crude percentages | Adjusted percentages | 95%C.I. lower limit | 95% C. I. upper limit |
| Not reported | blank | 172 | 6.40% | 5.42% | 2.10% | 8.74% | 34 | 0.00% | 0.00% | 0.00% | 0.00% | 55 | 7.30% | 5.60% | 0.30% | 10.90% |
| Members of legislative and governmental bodies, executives in public administration | 1.0 | 45 | 4.44% | 4.63% | 0.00% | 11.69% | 6 | 16.70% | 7.40% | 0.00% | 20.10% | 9 | 0.00% | 0.00% | 0.00% | 0.00% |
| Entrepreneurs, directors and managers of large companies | 1.1 | 85 | 9.41% | 5.68% | 1.15% | 10.20% | 15 | 0.00% | 0.00% | 0.00% | 0.00% | 22 | 9.10% | 4.20% | 0.00% | 9.70% |
| Entrepreneurs and managers of small businesses | 1.2 | 82 | 3.66% | 3.12% | 0.00% | 6.85% | 16 | 0.00% | 0.00% | 0.00% | 0.00% | 20 | 10.00% | 3.70% | 0.00% | 8.50% |
| Specialists in mathematical, computer, chemical, physical and natural sciences | 1.3 | 259 | 4.25% | 4.94% | 2.15% | 7.73% | 31 | 0.00% | 0.00% | 0.00% | 0.00% | 70 | 7.10% | 5.10% | 0.80% | 9.40% |
| Engineers, architects and assimilated professions | 2.1 | 259 | 4.25% | 4.06% | 1.59% | 6.53% | 34 | 5.90% | 9.50% | 0.00% | 23.00% | 23 | 0.00% | 0.00% | 0.00% | 0.00% |
| Specialists in the life sciences | 2.2 | 38 | 2.63% | 3.36% | 0.00% | 9.64% | 3 | 0.00% | 0.00% | 0.00% | 0.00% | 10 | 30.00% | 22.30% | 2.80% | 41.80% |
| Specialists in health care | 2.3 | 87 | 2.30% | 1.12% | 0.00% | 2.65% | 26 | 7.70% | 41.70% | 33.10% | 50.40% | 5 | 20.00% | 9.90% | 0.00% | 25.80% |
| Specialists in the humanities, social sciences, arts and management | 2.4 | 488 | 3.89% | 3.39% | 1.87% | 4.91% | 118 | 4.20% | 4.70% | 0.60% | 8.80% | 87 | 6.90% | 7.90% | 0.00% | 16.50% |
| Education and research specialists | 2.5 | 338 | 4.14% | 3.15% | 1.36% | 4.94% | 54 | 5.60% | 9.80% | 0.00% | 23.10% | 44 | 9.10% | 4.80% | 0.50% | 9.00% |
| Technical professions in science, engineering and manufacturing | 2.6 | 571 | 5.95% | 5.64% | 3.79% | 7.49% | 73 | 4.10% | 4.10% | 0.00% | 8.60% | 158 | 7.60% | 8.30% | 3.30% | 13.30% |
| Technical professions in organization, administration, and financial and business activities | 3.1 | 158 | 5.70% | 5.63% | 2.05% | 9.21% | 17 | 5.90% | 5.90% | 0.00% | 16.40% | 31 | 12.90% | 7.00% | 1.00% | 13.00% |
| Technical professions in health and life sciences | 3.2 | 463 | 6.91% | 6.48% | 4.26% | 8.70% | 80 | 8.80% | 8.30% | 2.40% | 14.20% | 100 | 7.00% | 5.50% | 1.00% | 10.00% |
| Technical professions in public and personal services | 3.3 | 162 | 1.23% | 1.54% | 0.00% | 3.93% | 35 | 2.90% | 3.30% | 0.00% | 9.40% | 33 | 18.20% | 16.10% | 0.80% | 31.50% |
| Secretarial and office machine clerks | 3.4 | 1198 | 4.51% | 4.96% | 3.66% | 6.27% | 241 | 10.00% | 10.90% | 6.70% | 15.00% | 223 | 8.50% | 9.10% | 4.90% | 13.40% |
| Clerical clerks in money movement and customer service | 4.1 | 121 | 1.65% | 1.19% | 0.00% | 2.82% | 47 | 2.10% | 2.40% | 0.00% | 6.90% | 22 | 4.50% | 2.10% | 0.00% | 6.10% |
| Administrative, accounting and financial management clerks | 4.2 | 250 | 3.60% | 3.75% | 1.34% | 6.15% | 70 | 2.90% | 3.70% | 0.00% | 8.90% | 43 | 11.60% | 14.20% | 2.50% | 25.80% |
| Clerks employed in the collection, control, storage and delivery of documentation | 4.3 | 71 | 5.63% | 3.99% | 0.04% | 7.94% | 20 | 0.00% | 0.00% | 0.00% | 0.00% | 13 | 7.70% | 5.00% | 0.00% | 13.80% |
| Skilled professions in commercial activities | 4.4 | 286 | 5.94% | 7.27% | 3.87% | 10.67% | 91 | 7.70% | 11.60% | 3.80% | 19.40% | 54 | 13.00% | 13.10% | 3.70% | 22.40% |
| Skilled professions in accommodation and food service activities | 5.1 | 196 | 2.55% | 7.89% | 0.81% | 14.96% | 99 | 2.00% | 4.10% | 0.00% | 11.00% | 20 | 0.00% | 0.00% | 0.00% | 0.00% |
| Skilled professions in health and social services | 5.2 | 17 | 0.00% | 0.00% | 0.00% | 0.00% | 3 | 33.30% | 33.30% | 33.30% | 33.30% | 2 | 0.00% | 0.00% | 0.00% | 0.00% |
| Skilled professions in cultural, security and personal services | 5.3 | 131 | 3.82% | 5.30% | 0.85% | 9.76% | 33 | 0.00% | 0.00% | 0.00% | 0.00% | 23 | 8.70% | 14.60% | 0.00% | 34.80% |
| Skilled craftsmen and workers in food processing, wood, textiles, clothing, leather, and entertainment industries | 5.4 | 148 | 4.05% | 5.33% | 0.86% | 9.81% | 30 | 0.00% | 0.00% | 0.00% | 0.00% | 22 | 4.50% | 3.30% | 0.00% | 9.40% |
| Craft and skilled metalworkers and installers and maintenance workers of electrical and electronic equipment | 6.1 | 124 | 2.42% | 4.13% | 0.00% | 8.94% | 25 | 0.00% | 0.00% | 0.00% | 0.00% | 18 | 5.60% | 2.30% | 0.00% | 6.60% |
| Craft and skilled metalworkers in precision mechanics, artistic handicrafts, printing and related trades | 6.2 | 136 | 5.88% | 6.65% | 2.20% | 11.10% | 23 | 4.30% | 2.80% | 0.00% | 8.00% | 16 | 6.30% | 2.70% | 0.00% | 7.80% |
| Farmers and skilled laborers in agriculture, forestry, animal husbandry, fishing and hunting | 6.3 | 74 | 1.35% | 2.56% | 0.00% | 7.38% | 38 | 2.60% | 5.00% | 0.00% | 13.80% | 18 | 16.70% | 6.90% | 0.10% | 13.70% |
| Craftsmen and skilled workers in 'mining, construction and maintenance of buildings | 6.4 | 89 | 3.37% | 6.40% | 0.00% | 14.12% | 36 | 5.60% | 8.50% | 0.00% | 18.50% | 15 | 13.30% | 7.40% | 0.00% | 16.40% |
| Industrial plant operators | 6.5 | 31 | 6.45% | 6.21% | 0.00% | 14.79% | 25 | 0.00% | 0.00% | 0.00% | 0.00% | 4 | 25.00% | 14.90% | 0.00% | 35.50% |
| Semi-skilled workers of fixed machinery for mass production and assembly workers | 7.1 | 47 | 2.13% | 1.75% | 0.00% | 5.08% | 27 | 3.70% | 5.90% | 0.00% | 16.40% | 4 | 25.00% | 33.30% | 33.30% | 33.30% |
| Stationary machinery operators in agriculture and the food industry | 7.2 | 17 | 0.00% | 0.00% | 0.00% | 0.00% | 2 | 0.00% | 0.00% | 0.00% | 0.00% | 4 | 0.00% | 0.00% | 0.00% | 0.00% |
| Drivers of vehicles, mobile and lifting machinery | 7.3 | 96 | 7.29% | 7.66% | 2.19% | 13.12% | 26 | 11.50% | 8.70% | 0.00% | 17.90% | 21 | 9.50% | 9.10% | 0.00% | 21.70% |
| Unskilled occupations in trade and services | 7.4 | 148 | 8.11% | 10.46% | 4.92% | 16.01% | 80 | 6.30% | 6.20% | 1.00% | 11.40% | 41 | 19.50% | 25.20% | 10.30% | 40.00% |
| Unskilled occupations in domestic, recreational and cultural activities | 8.1 | 16 | 0.00% | 0.00% | 0.00% | 0.00% | 14 | 21.40% | 20.30% | 0.00% | 41.30% | 5 | 20.00% | 16.60% | 0.00% | 39.70% |
| Unskilled professions in manufacturing, mineral extraction and construction | 8.2 | 12 | 8.33% | 9.92% | 0.00% | 25.78% | 10 | 0.00% | 0.00% | 0.00% | 0.00% | 2 | 50.00% | 29.70% | 29.70% | 29.70% |
| Unskilled professions in agriculture, grounds maintenance, animal husbandry, forestry and fishing | 8.3 | 103 | 5.83% | 5.13% | 1.08% | 9.18% | 15 | 0.00% | 0.00% | 0.00% | 0.00% | 38 | 10.50% | 6.70% | 0.40% | 13.00% |
| Officers in the armed forces | 9.1 | 6 | 0.00% | 0.00% | 0.00% | 0.00% | 1 | 0.00% | 0.00% | 0.00% | 0.00% | 2 | 0.00% | 0.00% | 0.00% | 0.00% |
| Sergeants, superintendents and marshals of the armed forces | 9.2 | 20 | 10.00% | 4.96% | 0.00% | 11.23% | 8 | 0.00% | 0.00% | 0.00% | 0.00% | 4 | 0.00% | 0.00% | 0.00% | 0.00% |
| Troops of the armed forces | 9.3 | 51 | 1.96% | 1.65% | 0.00% | 4.80% | 11 | 0.00% | 0.00% | 0.00% | 0.00% | 6 | 0.00% | 0.00% | 0.00% | 0.00% |

**Table A6. Crude and adjusted percentages, with 95% confidence interval (C.I.), of female workers in bad health by educational mismatch, gender and occupation**

| **Occupation** |  | Percentage of FEMALES in bad health / Adequate education | | | | | Percentage of Females in bad health / Over educated | | | | | Percentage of Females in bad health / Under educated | | | | |
| --- | --- | --- | --- | --- | --- | --- | --- | --- | --- | --- | --- | --- | --- | --- | --- | --- |
|  | Code | n (sample) | Crude percentages | Adjusted percentages | 95%C.I. lower limit | 95% C. I. upper limit | n (sample) | Crude percentages | Adjusted percentages | 95%C.I. lower limit | 95% C. I. upper limit | n (sample) | Crude percentages | Adjusted percentages | 95%C.I. lower limit | 95% C. I. upper limit |
| Not reported | blank | 229 | 3.93% | 3.83% | 1.36% | 6.30% | 56 | 8.90% | 9.00% | 1.10% | 16.90% | 51 | 13.70% | 13.90% | 4.00% | 23.80% |
| Members of legislative and governmental bodies, executives in public administration | 1.0 | 21 | 23.81% | 12.39% | 4.10% | 20.69% | 7 | 14.30% | 11.10% | 0.00% | 28.90% | 4 | 50.00% | 33.30% | 33.30% | 33.30% |
| Entrepreneurs, directors and managers of large companies | 1.1 | 25 | 0.00% | 0.00% | 0.00% | 0.00% | 8 | 0.00% | 0.00% | 0.00% | 0.00% | 9 | 22.20% | 18.50% | 0.40% | 36.60% |
| Entrepreneurs and managers of small businesses | 1.2 | 41 | 0.00% | 0.00% | 0.00% | 0.00% | 17 | 5.90% | 7.40% | 0.00% | 20.10% | 8 | 12.50% | 11.10% | 0.00% | 28.90% |
| Specialists in mathematical, computer, chemical, physical and natural sciences | 1.3 | 110 | 4.55% | 4.64% | 0.67% | 8.60% | 24 | 8.30% | 32.30% | 27.50% | 37.10% | 25 | 4.00% | 3.70% | 0.00% | 10.50% |
| Engineers, architects and assimilated professions | 2.1 | 132 | 5.30% | 2.68% | 0.78% | 4.58% | 30 | 23.30% | 30.70% | 8.50% | 52.90% | 10 | 0.00% | 0.00% | 0.00% | 0.00% |
| Specialists in the life sciences | 2.2 | 86 | 6.98% | 7.37% | 1.81% | 12.92% | 15 | 13.30% | 28.40% | 0.00% | 58.50% | 3 | 0.00% | 0.00% | 0.00% | 0.00% |
| Specialists in health care | 2.3 | 91 | 5.49% | 4.61% | 0.65% | 8.57% | 22 | 9.10% | 10.60% | 0.00% | 24.00% | 8 | 0.00% | 0.00% | 0.00% | 0.00% |
| Specialists in the humanities, social sciences, arts and management | 2.4 | 535 | 5.05% | 4.88% | 2.98% | 6.78% | 139 | 5.80% | 4.90% | 0.90% | 8.90% | 52 | 11.50% | 9.30% | 2.40% | 16.20% |
| Education and research specialists | 2.5 | 734 | 5.45% | 4.12% | 2.90% | 5.35% | 150 | 11.30% | 9.30% | 5.00% | 13.50% | 119 | 10.10% | 5.80% | 2.60% | 9.00% |
| Technical professions in science, engineering and manufacturing | 2.6 | 225 | 5.78% | 5.76% | 2.33% | 9.19% | 41 | 4.90% | 14.90% | 0.30% | 29.40% | 34 | 8.80% | 10.50% | 0.00% | 21.10% |
| Technical professions in organization, administration, and financial and business activities | 3.1 | 397 | 6.80% | 9.35% | 5.66% | 13.05% | 62 | 9.70% | 15.90% | 0.00% | 32.50% | 48 | 6.30% | 10.70% | 0.00% | 22.10% |
| Technical professions in health and life sciences | 3.2 | 380 | 6.84% | 7.16% | 4.45% | 9.87% | 71 | 7.00% | 8.10% | 1.10% | 15.10% | 67 | 9.00% | 7.70% | 2.00% | 13.50% |
| Technical professions in public and personal services | 3.3 | 211 | 3.32% | 3.37% | 0.88% | 5.86% | 53 | 11.30% | 9.50% | 1.90% | 17.10% | 43 | 4.70% | 4.50% | 0.00% | 10.50% |
| Secretarial and office machine clerks | 3.4 | 1755 | 7.86% | 7.78% | 6.53% | 9.03% | 448 | 7.60% | 9.00% | 6.00% | 12.00% | 226 | 14.20% | 12.10% | 7.90% | 16.20% |
| Clerical clerks in money movement and customer service | 4.1 | 139 | 10.79% | 11.80% | 6.07% | 17.53% | 71 | 4.20% | 2.30% | 0.00% | 4.90% | 18 | 16.70% | 11.10% | 0.80% | 21.30% |
| Administrative, accounting and financial management clerks | 4.2 | 205 | 7.32% | 8.15% | 4.23% | 12.07% | 56 | 5.40% | 2.40% | 0.00% | 5.10% | 19 | 5.30% | 3.70% | 0.00% | 10.50% |
| Clerks employed in the collection, control, storage and delivery of documentation | 4.3 | 69 | 8.70% | 6.73% | 1.44% | 12.02% | 22 | 9.10% | 4.80% | 0.00% | 10.90% | 19 | 10.50% | 8.90% | 0.00% | 20.50% |
| Skilled professions in commercial activities | 4.4 | 366 | 6.01% | 7.99% | 4.82% | 11.15% | 238 | 2.50% | 7.30% | 1.50% | 13.10% | 50 | 12.00% | 10.00% | 2.70% | 17.20% |
| Skilled professions in accommodation and food service activities | 5.1 | 223 | 2.69% | 4.82% | 0.39% | 9.24% | 162 | 0.00% | 0.00% | 0.00% | 0.00% | 25 | 12.00% | 9.90% | 0.00% | 20.80% |
| Skilled professions in health and social services | 5.2 | 28 | 7.14% | 16.64% | 0.33% | 32.95% | 5 | 0.00% | 0.00% | 0.00% | 0.00% | 8 | 0.00% | 0.00% | 0.00% | 0.00% |
| Skilled professions in cultural, security and personal services | 5.3 | 186 | 9.68% | 12.38% | 6.54% | 18.23% | 71 | 8.50% | 11.20% | 3.20% | 19.10% | 32 | 34.40% | 34.00% | 16.90% | 51.00% |
| Skilled craftsmen and workers in food processing, wood, textiles, clothing, leather, and entertainment industries | 5.4 | 27 | 3.70% | 4.96% | 0.00% | 13.83% | 17 | 5.90% | 4.20% | 0.00% | 11.80% | 5 | 20.00% | 9.90% | 0.00% | 25.80% |
| Craft and skilled metalworkers and installers and maintenance workers of electrical and electronic equipment | 6.1 | 24 | 4.17% | 11.10% | 0.00% | 28.85% | 4 | 25.00% | 11.10% | 0.00% | 28.90% | 3 | 0.00% | 0.00% | 0.00% | 0.00% |
| Craft and skilled metalworkers in precision mechanics, artistic handicrafts, printing and related trades | 6.2 | 24 | 8.33% | 11.90% | 0.00% | 24.67% | 8 | 0.00% | 0.00% | 0.00% | 0.00% | 3 | 0.00% | 0.00% | 0.00% | 0.00% |
| Farmers and skilled laborers in agriculture, forestry, animal husbandry, fishing and hunting | 6.3 | 28 | 10.71% | 12.19% | 0.00% | 24.61% | 23 | 17.40% | 17.70% | 1.30% | 34.00% | 5 | 0.00% | 0.00% | 0.00% | 0.00% |
| Craftsmen and skilled workers in 'mining, construction and maintenance of buildings | 6.4 | 46 | 6.52% | 6.24% | 0.00% | 12.61% | 24 | 8.30% | 13.30% | 0.00% | 30.00% | 9 | 0.00% | 0.00% | 0.00% | 0.00% |
| Industrial plant operators | 6.5 | 17 | 0.00% | 0.00% | 0.00% | 0.00% | 11 | 9.10% | 16.60% | 0.00% | 39.70% | 2 | 0.00% | 0.00% | 0.00% | 0.00% |
| Semi-skilled workers of fixed machinery for mass production and assembly workers | 7.1 | 16 | 18.75% | 8.92% | 0.48% | 17.37% | 12 | 8.30% | 14.90% | 0.00% | 35.50% | 9 | 22.20% | 11.90% | 0.00% | 24.70% |
| Stationary machinery operators in agriculture and the food industry | 7.2 | 9 | 22.22% | 9.24% | 0.00% | 20.33% | 3 | 0.00% | 0.00% | 0.00% | 0.00% | 1 | 0.00% | 0.00% | 0.00% | 0.00% |
| Drivers of vehicles, mobile and lifting machinery | 7.3 | 11 | 0.00% | 0.00% | 0.00% | 0.00% | 5 | 40.00% | 45.30% | 31.20% | 59.40% | 2 | 0.00% | 0.00% | 0.00% | 0.00% |
| Unskilled occupations in trade and services | 7.4 | 135 | 12.59% | 12.39% | 6.95% | 17.83% | 88 | 10.20% | 11.60% | 4.50% | 18.60% | 31 | 22.60% | 22.40% | 7.80% | 37.00% |
| Unskilled occupations in domestic, recreational and cultural activities | 8.1 | 53 | 16.98% | 17.31% | 7.76% | 26.86% | 19 | 5.30% | 3.30% | 0.00% | 9.50% | 16 | 25.00% | 17.20% | 0.80% | 33.70% |
| Unskilled professions in manufacturing, mineral extraction and construction | 8.2 | 10 | 10.00% | 14.87% | 0.00% | 35.48% | 5 | 0.00% | 0.00% | 0.00% | 0.00% | 2 | 0.00% | 0.00% | 0.00% | 0.00% |
| Unskilled professions in agriculture, grounds maintenance, animal husbandry, forestry and fishing | 8.3 | 40 | 7.50% | 8.72% | 0.00% | 17.75% | 8 | 25.00% | 11.90% | 0.00% | 24.70% | 7 | 14.30% | 11.10% | 0.00% | 28.90% |
| Officers in the armed forces | 9.1 | 5 | 0.00% | 0.00% | 0.00% | 0.00% | 0 | 0.00% | 0.00% | 0.00% | 0.00% | 0 | 0.00% | 0.00% | 0.00% | 0.00% |
| Sergeants, superintendents and marshals of the armed forces | 9.2 | 3 | 0.00% | 0.00% | 0.00% | 0.00% | 0 | 0.00% | 0.00% | 0.00% | 0.00% | 0 | 0.00% | 0.00% | 0.00% | 0.00% |
| Troops of the armed forces | 9.3 | 14 | 0.00% | 0.00% | 0.00% | 0.00% | 6 | 0.00% | 0.00% | 0.00% | 0.00% | 2 | 0.00% | 0.00% | 0.00% | 0.00% |

**Figure A1 Vertical Mismatch**


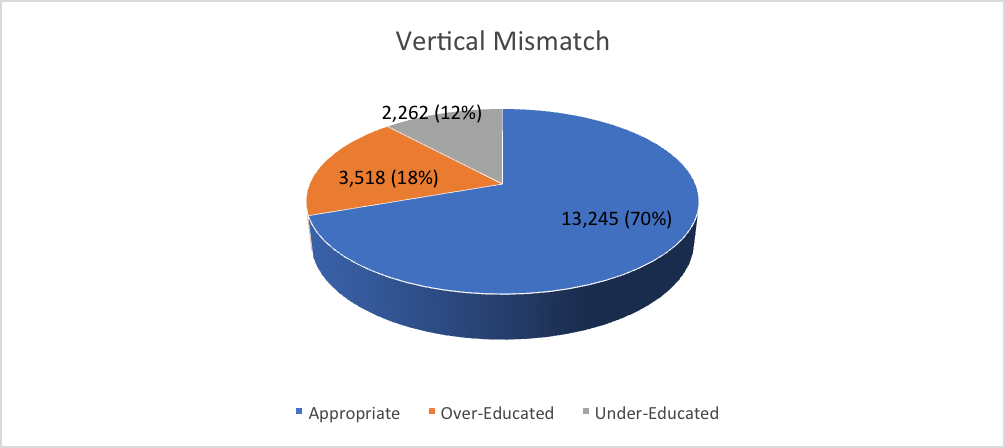


**Figure A2 Perceived health by educational mismatch**


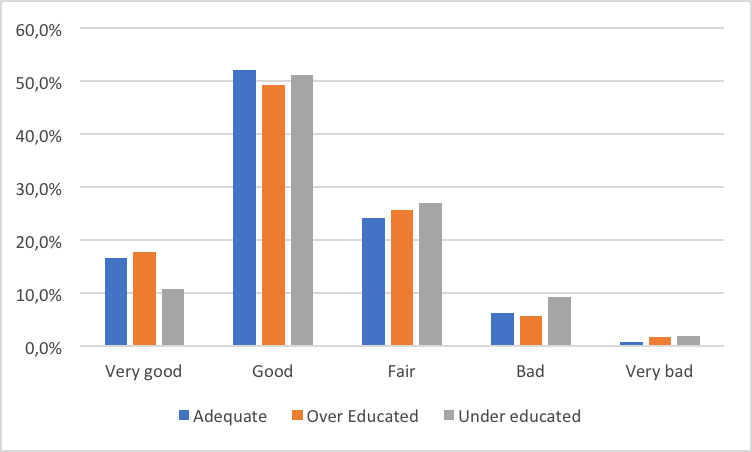

Supplement: Supplementary file 1 [file Data_Sheet_1.doc]
